# Association between non-alcoholic fatty liver disease and metabolically healthy deterioration across different body shape phenotypes at baseline and change patterns

**DOI:** 10.1038/s41598-022-18988-x

**Published:** 2022-08-30

**Authors:** Liu Lei, Wang Changfa, Wang Jiangang, Chen Zhiheng, Yuan Ting, Zhu Xiaoling, Deng Yuling, Wang Yaqin

**Affiliations:** 1 Health Management Center, The Third Xiangya Hospital, Central South University, No. 138 Tongzipo Road, Yuelu District, Changsha, 410013 Hunan China; 2 General Surgery Department, The Third Xiangya Hospital, Central South University, No.138 Tongzipo Road, Yuelu District, Changsha, 410013 Hunan China

**Keywords:** Obesity, Risk factors

## Abstract

Non-alcoholic fatty liver disease (NAFLD) is a hepatic manifestation of metabolic syndrome (MetS), and the relationship between NAFLD and metabolic deterioration remains unclear. This study aimed to investigate dynamic changes in metabolically healthy phenotypes and to assess the impact of non-alcoholic fatty liver disease (NAFLD) on the conversion from metabolically healthy (MH) to metabolically unhealthy (MU) phenotypes across body shape phenotypes and phenotypic change patterns. We defined body shape phenotypes using both the body mass index (BMI) and waist circumference (WC) and defined metabolic health as individuals scoring ≤ 1 on the NCEP-ATP III criteria, excluding WC. A total of 12,910 Chinese participants who were MH at baseline were enrolled in 2013 and followed-up in 2019 or 2020. During a median follow-up of 6.9 years, 27.0% (n = 3,486) of the MH individuals developed an MU phenotype. According to the multivariate Cox analyses, NAFLD was a significant predictor of conversion from the MH to MU phenotype, independent of potential confounders (HR: 1.12; 95% confidence interval: 1.02–1.22). For the MH-normal weight group, the relative risk of NAFLD in phenotypic conversion was 1.21 (95% CI 1.03–1.41, *P* = 0.017), which was relatively higher than that of MH-overweight/obesity group (HR: 1.14, 95% CI 1.02–1.26, *P* = 0.013). Interestingly, the effect of NAFLD at baseline on MH deterioration was stronger in the “lean” phenotype group than in the “non-lean” phenotype group at baseline and in the “fluctuating non-lean” phenotype change pattern group than in the “stable non-lean” phenotype change pattern group during follow-up. In conclusion, lean NAFLD is not as benign as currently considered and requires more attention during metabolic status screening.

## Introduction

In China, an obesity epidemic is occurring alongside rapid socioeconomic development, which has become a major challenge to national public health^1^. According to the latest national statistics, 16.4% of adults are obese, and 34.3% qualify as overweight based on Chinese standards^2^. Obesity-associated metabolic disorders such as hyperglycemia, hypertension and hypercholesterolemia increase the risk of cardiovascular disease (CVD), which is currently the leading cause of disability and death among Chinese adults^3,4^. With advancements in obesity research, researchers have gradually recognized that not all obesity is accompanied by metabolic abnormalities, resulting in the formation of the metabolically healthy obesity (MHO) and metabolically unhealthy obesity (MUO) categories^5,6^. Nevertheless, the CVD risk in MHO is higher in studies that have longer follow-up periods, as more than one-third of individuals with MHO later develop metabolic abnormalities or diabetes, which suggests that the MHO phenotype is a transient condition^7^. Therefore, identifying risk factors for the progression of metabolically healthy (MH) deterioration is important for clinical settings.

Body mass index (BMI), used to categorize overall obesity, and waist circumference (WC), which refers to abdominal obesity, are common measures of obesity in clinical guidelines. Chinese adults have a higher proportion of body fat and a greater propensity for central adiposity than their Western counterparts^8^. However, measuring body shape at an initial or a single time point, ignoring potential change pattern in body shape over time, may underestimate health risks. An increasing number of studies have focused on the relationship between body shape change patterns and chronic diseases, such as CVD and diabetes^9^. Although BMI and WC are acceptable measures for overall and abdominal adiposity, they do not capture information about body fat distribution, such as ectopic fat^10^. Ectopic fat depots (in the liver, heart or pancreas) are associated with cardiometabolic risk, and visceral adiposity might account for the high cardiometabolic risk of individuals with low BMI^11,12^. Non-alcoholic fatty liver disease (NAFLD), considered to be a hepatic manifestation of metabolic syndrome (MetS), is the leading cause of chronic liver disease; its prevalence has gradually increased worldwide along with the global obesity epidemic^13,14^. Accumulating evidence has confirmed that NAFLD is associated with insulin resistance, type 2 diabetes, CVD and cardiovascular mortality^15^.

Some studies have found that BMI, visceral abdominal fat (VAT) and subcutaneous abdominal fat (SAT) measured directly by computed tomography (CT), and the visceral adipose index (VAI) predict MH deterioration in obese or non-obese individuals^12,16,17^. However, most research has focused only on a single-time assessment of body shape to predict MH conversion, ignoring the risk factors for transition in the different body shape change pattern subgroups. In addition, few studies have addressed the risk factors for MH transitions in the Chinese population. In our study, we observed 12,910 Chinese individuals for a median follow-up of 6.9 years and aimed to determine how frequently MH phenotypes converted to metabolically unhealthy (MU) phenotypes and which demographic, lifestyle, clinical, and metabolic variables predict this conversion in different body shape change pattern subgroups, with a particular focus on the roles of NAFLD in this conversion.

## Materials and methods

### Subjects

Participants were recruited from the Health Management Institution of Third Xiangya Hospital of Central South University (Changsha), the largest medical institution in Central China. A total of 19,128 subjects aged 18 to 65 years who received health check-up examinations participated in the prospective study, and data collected in 2013 were used as the baseline sample. The second survey was repeated in 2019 or 2020 with a median follow-up of 6.9 years. No participants participated in special weight loss programs, including lifestyle or drug weight loss programs. Individuals who were underweight at baseline (BMI < 18.5 kg/m^2^) were initially excluded. Secondly, we excluded individuals who met any of the following criteria: (1) severe renal dysfunction (n = 78); (2) malignant tumors (n = 169); (3) CVDs, such as myocardial infarction, angina pectoris, heart failure, stroke or transient ischemic attack (n = 79); (4) pregnancy (n = 33); (5) hepatitis B surface antigen positivity (n = 298); (6) hepatitis C antibody positivity (n = 36); (7) alcohol consumption ≥ 30 g/d in men or ≥ 20 g/d in women (n = 134); and (8) missing anthropometric or metabolic data (n = 784). Of the remaining subjects, we selected those who were MH status at baseline. Ultimately, 12,910 participants were included. The study protocol (Ethics approval code: 2013BAI04B01) was approved by the Ethics Committee of Central South University, and this research was performed in accordance with the Declaration of Helsinki. Written informed consent was obtained from all participants.

### Demographic and clinical characteristics

The same measurements were conducted at both the baseline and follow-up assessments. Data on basic demographic and clinical characteristics, such as age, sex, marital status, educational level, employment status and lifestyle, were collected through standardized health questionnaires presented via a website (https://new.selfhealth.com.cn/#/login). Additionally, we collected data on the medical history and personal history of the participants.

Cigarette smoking was defined as having smoked ≥ 100 cigarettes in one’s lifetime and a current smoking habit. Alcohol consumption was coded as present if the individual reported the consumption of beer, wine (including Chinese wine) and/or liquor at least two days per week over a period exceeding 12 months. Regular leisure-time physical activity was defined as performing ≥ 30 min per day of moderate or vigorous activity for ≥ 3 d/week^18^. Insufficient sleep was defined as sleeping < 7 h per day^19^.

### Clinical and laboratory measurements

Height and weight were measured on a scale, with the subjects wearing light clothing and no shoes. BMI was calculated as the weight in kilograms divided by the height in meters squared. WC was measured twice halfway between the lowest rib and the top of the pelvis without applying pressure to the body surface after a normal expiration. The mean of the two measurements was calculated. Blood pressure (BP) was measured on the right upper arm in the sitting position after 10–15 min of rest using a validated digital automatic analyzer (Omron 9020). Systolic BP and diastolic BP were each measured twice, and the mean of the two readings was used in the analysis.

After a 12-h overnight fast, fasting venous blood samples were collected and immediately processed and analyzed at the clinical laboratory of Third Xiangya Hospital. The sample analysis was performed in accordance with the manufacturer’s specifications. Fasting blood glucose was measured with the glucose oxidase method, and we determined high-density lipoprotein (HDL) cholesterol, low-density lipoprotein (LDL) cholesterol, and levels of triglycerides, creatinine (Scr), blood urea nitrogen (BUN) and uric acid with enzymatic methods. Alanine transaminase (ALT) and albumin levels were determined by the bromocresol green (BCG) method. These analyses performed by an automated analyzer (Hitachi 7600–110; Hitachi, Tokyo, Japan). Renal disease was defined according to an estimated glomerular filtration rate (eGFR) < 60 ml/min/1.73 m^2^, calculated using the Modification of Diet in Renal Disease formula with an ethnicity coefficient for Chinese subjects as follows: eGFR = 175 × Scr^−1.234^ × age^−0.179^[if female, × 0.79]^20^.

### Definitions

We classified participants into the following BMI categories based on the Working Group on Obesity in China criteria: normal weight (NW) (BMI 18.5–23.9 kg/m^2^), overweight (OW) (BMI 24.0–27.9 kg/m^2^), and obesity (OB) (BMI ≥ 28 kg/m^2^)^2^. The prevalence rates of NW, OW, and OB were 54.9% (n = 9,609), 35.6% (n = 6,242), and 9.5% (n = 1,666), respectively. Because of the relatively limited number of obese subjects in this cohort, we divided subjects into two body shape groups: NW (lean) vs. OW/OB (non-lean) groups. Sex-specific abdominal obesity was defined according to the Asian-Pacific Guidelines using the national WC cutoff points: a WC ≥ 90 cm for men and ≥ 85 cm for women^21^. We used the National Cholesterol Education Program-Adult Treatment Panel III definition of MetS to determine the MU phenotype (WC was not included in the definition of metabolic health because of collinearity with BMI): (1) hypertriglyceridemia, defined as a triglyceride (TG) level ≥ 1.69 mmol/L and/or the use of lipid-lowering drugs;  (2) low HDL cholesterol, defined as a HDL cholesterol level < 1.03 mmol/L in men and < 1.29 mmol/L in women; (3) elevated BP, defined as a systolic BP ≥ 130 mmHg and/or a diastolic BP ≥ 85 mmHg and/or the use of antihypertensive drugs and/or a self-reported history of hypertension; and  (4) hyperglycemia, defined as a blood glucose level ≥ 5.6 mmol/L and/or the use of any medications for diabetes (insulin or oral glucose-lowering medications) and/or a self-reported history of diabetes. Individuals exhibiting one or fewer of these components were deemed MH, whereas MU individuals were defined as those displaying at least two of these components^22^. The definitions of hypertension, diabetes and the prevalence of medication history are detailed in Supplemental Table 1.

Based on the combination of BMI categories and MH status, participants were then categorized into the following 4 groups: metabolically healthy normal weight (MH-NW); metabolically healthy overweight/obesity (MH-OW/OB); metabolically unhealthy normal weight (MU-NW); and metabolically unhealthy overweight/obesity (MU-OW/OB).

### Ascertainment of body shape change patterns

The BMI/WC percent change (ΔBMI/WC) was calculated between follow-up and baseline ((the value at the second survey − the value at baseline)/the value at baseline × 100%). BMI and WC percent change patterns were each classified into 3 groups, including loss, maintenance and gain, which were defined as percent changes of < − 3%, − 3% ≤ percent changes ≤ 3% and percent changes > 3%, respectively^23^.

In addition, we defined 2 × 4 groups based on BMI and WC status change patterns: (1) the BMI-stable normal group (BMI < 24.0 at both baseline and second survey), the normal to general overweight/obesity group (BMI < 24.0 at baseline and BMI ≥ 24.0 at second survey), the general overweight/obesity to normal group (BMI ≥ 24.0 at baseline and BMI < 24.0 at second survey), and the stable general overweight/obesity group (BMI ≥ 24.0 at both baseline and second survey); and (2) the WC-stable normal group (< 90 cm for men and < 85 cm for women at both baseline and second survey), the normal to abdominal obesity group (normal at baseline and ≥ 90 cm for men and ≥ 85 cm for women at second survey), the abdominal obesity to normal group (≥ 90 cm for men and ≥ 85 cm for women at baseline and normal at second survey), and the stable abdominal obesity group (≥ 90 cm for men and ≥ 85 cm for women at both baseline and second survey).

### Ascertainment of fatty liver

Abdominal ultrasonography (with a Siemens AcusonSequoiaTM512 Ultrasound System (Mountain View, CA, USA)) was performed by clinical radiologists who were blinded to the subjects’ clinical diagnoses and biochemical tests. Positive abdominal ultrasound images included a diffusely increased liver near-field ultrasound echo (‘bright liver’), a liver echo greater than that of the kidney, vascular blurring and the gradual attenuation of far-field ultrasound echo^24^. Subjects with at least two of the abnormal findings listed above were defined as having hepatic steatosis^25^. NAFLD was diagnosed once the other causes of liver disease, especially excessive alcohol consumption, autoimmune hepatitis and the ingestion of drugs known to produce hepatic steatosis in the previous months, were rigorously excluded.

### Statistical analysis

Continuous variables are reported as the means ± standard deviations, and categorical variables are presented as percentages when appropriate. Descriptive statistics were used to summarize the baseline characteristics of the participants by the metabolic status groups. Chi-square tests and Mann–Whitney tests or t tests were used to assess the significance of differences in the categorical and continuous variables, respectively.

The time of follow-up was calculated from baseline to the end of follow-up. Univariate and multivariate Cox proportional hazard models were used with the time of follow-up as the time scale to estimate the hazard ratios (HRs) for incident conversion from the MH to the MU phenotype. The proportional hazard assumption was examined by Schoenfeld residuals. If variables considered for adjustment were found to have nonproportional hazards, we used Cox regression models with time-dependent coefficients for those variables. To prevent multicollinearity among different covariates in the model, we excluded variables with a variance inflation factor > 5. Only non-multicollinear variables and significant variables according to the univariate Cox model were included in the multivariate Cox models.

We further examined the relationship between NAFLD and the conversion from the MH to the MU phenotype according to baseline BMI (2 groups) and baseline WC (2 groups) or the BMI and WC change patterns across the follow-up period (2 × 3 percent change groups and 2 × 4 phenotype change pattern groups, respectively). The HRs and corresponding 95% CIs were also calculated by Cox proportional hazard regression analyses adjusted for age and sex. *P* < 0.05 was considered statistically significant.

## Results

### Baseline characteristics of the study subjects and body shape change patterns across the follow-up period

Table 1 presents the participants’ baseline characteristics stratified by BMI categories. Among the 12,910 participants, the mean (SD) age was 39.2 (10.2) years, 46.2% (n = 5,967) were men and 34.3% (n = 4,432) were overweight/obese. Within the BMI category of OW/OB, the participants had worse metabolic parameters (higher WC, blood pressure, and lipid, fasting glucose, uric acid, BUN, creatinine and ALT levels), had unfavorable lifestyle factors (current smoking habits, greater alcohol consumption, less physical activity and insufficient sleep), were more likely to have NAFLD and were older and less educated than those in the MH-NW category (all *P* < 0.002). The MH-NW participants had greater gains in BMI and WC than their MH-OW/OB counterparts. The proportion of individuals in each body shape change pattern group significantly differed between the MH-NW and MH-OW/OB groups at baseline.

**Table 1:** Baseline characteristics of the study population and body shape change patterns across the follow-up.

Variable	Metabolically healthy			*P* value
	All	MH–NW	MH–OW/OB	
**Number of subjects**	12,910	8478	4432	
Age, years	39.2 ± 10.2	38.0 ± 10.2	41.6 ± 9.86	< 0.001
Male sex, n (%)	5967 (46.2)	2805 (33.1)	3162 (71.3)	< 0.001
Being married, n (%)	11,718 (90.8)	7687 (90.7)	4031 (91.0)	0.599
University degree (education), n (%)	8875 (68.7)	5904 (69.6)	2971 (67.0)	0.002
Workers (occupation), n (%)	9598 (74.3)	6296 (74.5)	3302 (74.3)	0.766
**Anthropometry**				
Waist circumference, cm	78.3 ± 9.3	73.6 ± 6.6	87.2 ± 6.9	< 0.001
BMI, kg/m^2^	23.0 ± 2.9	21.3 ± 1.6	26.2 ± 1.9	< 0.001
Systolic blood pressure, mmHg	115.5 ± 12.5	112.9 ± 11.9	120.4 ± 12.2	< 0.001
Diastolic blood pressure, mmHg	71.3 ± 9.3	69.5 ± 8.8	74.7 ± 9.4	< 0.001
**Lifestyle factors**				
Current smoking, n (%)	2713 (21.0)	1311 (15.5)	1402 (31.6)	< 0.001
Alcohol drinking, n (%)	2920 (22.6)	1454 (17.2)	1466 (33.1)	< 0.001
Regular physical activity, n (%)	4364 (31.4)	2658 (38.5)	1706 (33.8)	< 0.001
Insufficient sleep, n (%)	1275 (9.9)	787 (9.3)	488 (11.0)	< 0.001
**Emerging risk factors and other**				
LDL–C, mmol/L	2.39 ± 0.76	2.25 ± 0.73	2.64 ± 0.75	< 0.001
Triglycerides, mmol/L	1.05 (0.77 − 1.43)	0.93 (0.71 − 1.25)	1.32 (0.99 − 1.70)	< 0.001
HDL–C, mmol/L	1.70 ± 0.39	1.79 ± 0.38	1.51 ± 0.32	< 0.001
Fasting glucose, mmol/L	5.03 ± 0.61	4.9 ± 0.60	5.1 ± 0.61	< 0.001
Uric acid, μmol/L	268.8 ± 85.0	244.6 ± 75.4	315.2 ± 83.1	< 0.001
BUN, mmol/L	4.39 ± 1.14	4.26 ± 1.12	4.64 ± 1.14	< 0.001
Creatinine, mmol/L	65.8 ± 15.1	62.5 ± 14.2	72.2 ± 14.7	< 0.001
Estimated GFR (mL/min)	122.5 ± 26.6	127.1 ± 27.9	113.8 ± 24.9	< 0.001
ALT, U/L	19.0 (14.0 − 27.0)	16.0 (12.0 − 22.0)	26.0 (18.0 − 37.0)	< 0.001
Albumin, g/L	47.0 ± 2.4	47.0 ± 2.4	47.0 ± 2.4	0.601
Total bilirubin, g/L	15.0 (12.2 − 18.5)	15.0 (12.2 − 18.5)	15.0 (12.2 − 18.4)	0.929
NAFLD, n (%)	2694 (20.9)	549 (6.5)	2145 (48.4)	< 0.001
**ΔBMI value change, kg/m**^**2**^	0.56 ± 0.01	0.80 ± 0.01	0.12 ± 0.02	< 0.001
**ΔWC value change, cm**	1.67 ± 0.05	2.10 ± 0.06	0.85 ± 0.09	< 0.001
**ΔBMI percent change categories (n (%))**				< 0.001
Maintenance (≥ –3% to ≤ 3%), n (%)	4792 (37.1)	2944 (34.7)	1848 (41.7)	
Gain (> 3%), n (%)	5882 (45.6)	4456 (52.6)	1426 (32.2)	
Loss (< –3%), n (%)	2236 (17.3)	1078 (12.7)	1158 (26.1)	
**ΔWC percent change categories (n (%))**				< 0.001
Maintenance (≥ –3% to ≤ 3%), n (%)	4411 (34.2)	2825 (33.3)	1586 (35.8)	
Gain (> 3%), n (%)	5741 (44.5)	4036 (47.6)	1705 (38.5)	
Loss (< –3%), n (%)	2758 (21.4)	1617 (19.1)	1141 (25.7)	
**BMI–status change categories (n (%))**				< 0.001
Stable normal	7118 (55.1)	7118 (84.0)	0	
Normal to general overweight/obesity	1360 (10.5)	1360 (16.0)	0	
General overweight/obesity to normal	606 (4.7)	0	606 (13.7)	
stable general overweight/obesity	3826 (29.6)	0	3826 (86.3)	
**WC–status change categories (n (%))**				< 0.001
Stable normal	9803 (75.9)	7956 (93.8)	1847 (41.7)	
Normal to abdominal obesity	1171 (9.1)	409 (4.8)	762 (17.2)	
Abdominal obesity to normal	519 (4.0)	62 (0.7)	457 (10.3)	
Stable abdominal obesity	1417 (11.0)	51 (0.6)	1366 (30.8)	

Values are expressed as means ± standard deviation, medians (interquartile range 25–75), or percentages. △BMI/WC value change between baseline and follow-up were expressed as mean ± standard error.

*WC* waist circumference, *BMI* body mass index, *LDL-C* low-density lipoprotein cholesterol, *HDL-C* high-density lipoprotein cholesterol, *BUN* Blood urea nitrogen, *GFR* glomerular filtration rate, *ALT* alanine transaminase, *NAFLD* non–alcoholic fatty liver disease.

### Prevalence of the conversion from MH to MU in the different body shape subgroups

**Figure 1:**
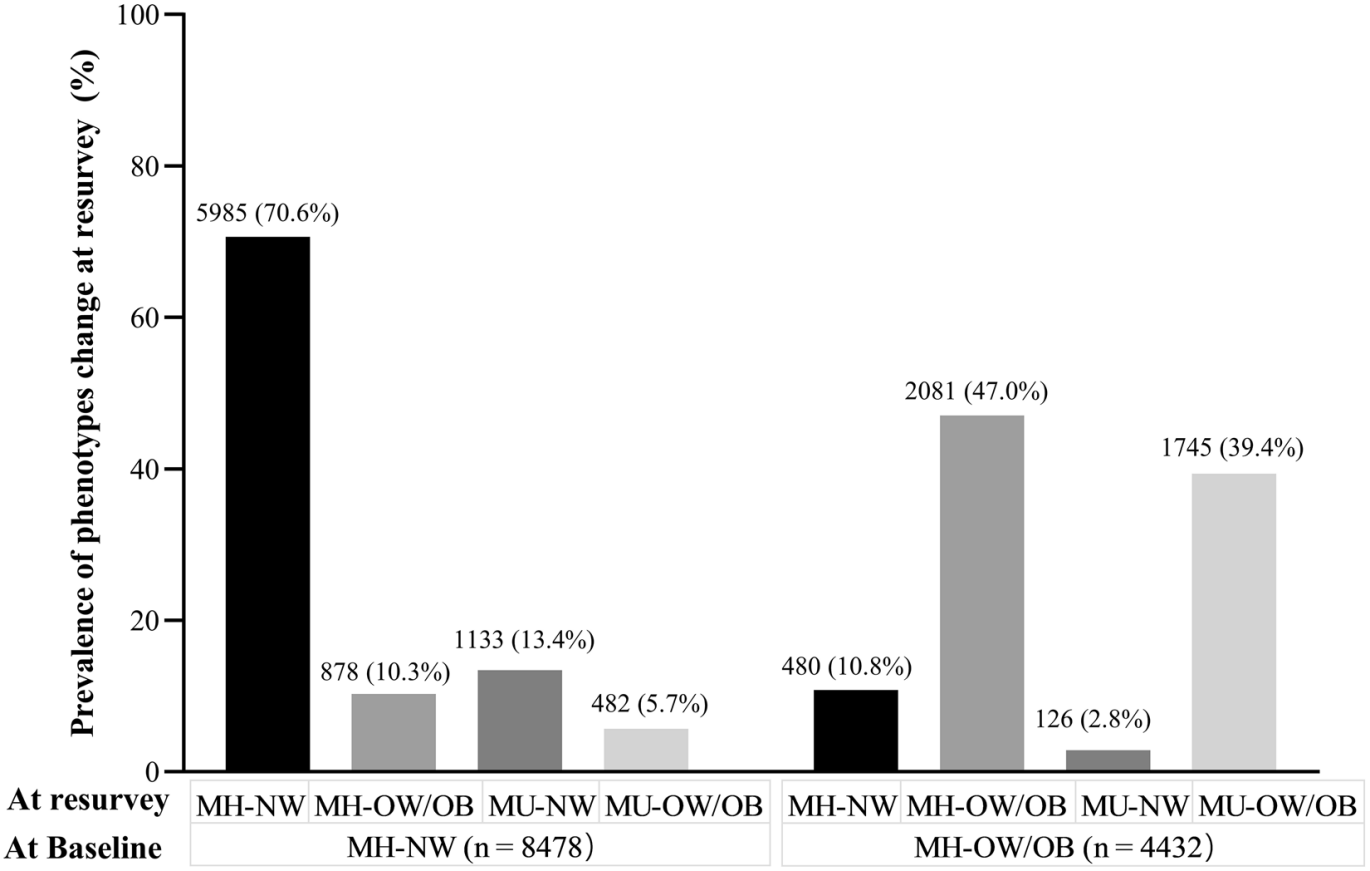
Prevalence of metabolic-BMI phenotypes at baseline and phenotypic transitions at second survey. The percentage shows the proportion of phenotypic transitions in the MH-NW and MH-OW/OB groups. MH-NW: metabolically healthy, normal weight; MH-OW/OB: metabolically healthy, overweight/obesity; MU-NW: metabolically unhealthy, normal weight; MU-OW/OB: metabolically unhealthy, overweight/obesity.

Figure 1 depicts the metabolic-BMI phenotype transitions over the observed period. Over a median 6.9-year follow-up, 27.0% (n = 3,486) of the MH individuals transitioned to the MU phenotype: 19.1% (n = 1,615) in the MH-NW group and 42.2% (n = 1,871) in the MH-OW/OB group. In addition, among individuals who were MH-NW at baseline, 70.6% remained in this category, and 10.3% gained weight and transitioned to the MH-OW/OB phenotype; among individuals who were MH-OW/OB at baseline, 47.0% remained in the same category, and 10.8% lost weight and progressed to the MH-NW phenotype over follow-up.

### Risks of the conversion from MH to MU according to Cox analyses

Tables 2 and 3 show the risks associated with deterioration from the MH to MU phenotype over time. In univariate Cox analyses, positive associations were observed between this deterioration and age, males, current smoking, individual components of MetS except for HDL cholesterol levels, renal function and uric acid levels, and a significantly high risk for the presence of NAFLD (HR: 2.19; 95% CI 2.04–2.34; *P* < 0.001) (Table 2). On the other hand, inverse associations emerged between this deterioration and individuals with a university degree, who worked, performed regular physical activity, and had higher HDL cholesterol and total bilirubin levels (Table 2). In multivariate Cox analyses (fully adjusted), NAFLD was a predictor of the conversion from the MH to the MU phenotype, independent of potential confounders (HR: 1.12; 95% CI 1.02–1.22; *P* = 0.014) (Table 3). For the MH-NW group, the relative risk of NAFLD predicted phenotypic conversion at HR = 1.21 (95% CI 1.03–1.41, *P* = 0.017), which was relatively higher than that of the MH-OW/OB group (HR: 1.14, 95% CI 1.02–1.26, *P* = 0.013) (Table 3).

**Table 2:** Univariate Cox analysis for transition from metabolically healthy to unhealthy phenotype over follow–up.

Variable	Metabolically healthy (ALL)	
	HR (95% CI)	*P* value
**Demographic features**		
Age, years	1.35 (1.29–1.40)	< 0.001
Sex, males	1.64 (1.53–1.75)	< 0.001
Marriage, married	1.07 (0.94–1.20)	0.308
Education, university degree	0.66 (0.61–0.70)	< 0.001
Occupation, worker	0.82 (0.76–0.88)	< 0.001
**Anthropometry**		
WC, cm	1.05 (1.04–1.05)	< 0.001
BMI, kg/m^2^	1.14 (1.13–1.16)	< 0.001
Systolic blood pressure, mmHg	1.16 (1.04–1.29)	0.008
Diastolic blood pressure, mmHg	1.26 (1.08–1.46)	0.003
**Lifestyle factors**		
Current smoking	1.38 (1.29–1.49)	< 0.001
Alcohol drinking	1.07 (0.99–1.15)	0.077
Regular physical activity	0.93 (0.84–0.97)	0.004
Insufficient sleep, < 7 h per day	1.09 (0.98–1.20)	0.125
**Emerging risk factors and other**		
LDL cholesterol, mmol/L	1.27 (1.22–1.32)	< 0.001
Triglycerides, mmol/L	1.33 (1.31–1.35)	< 0.001
HDL cholesterol, mmol/L	0.25 (0.22–0.28)	< 0.001
Fasting glucose, mmol/L	1.26 (1.23–1.30)	< 0.001
Uric acid, μmol/L	1.02 (1.01–1.03)	< 0.001
Blood urea nitrogen, mmol/L	1.12 (1.09–1.15)	< 0.001
Creatinine, mmol/L	1.02 (1.01–1.03)	< 0.001
Estimated GFR, mL/min	0.98 (0.97–0.99)	< 0.001
ALT, U/L	1.02(1.01–1.03)	< 0.001
Albumin, g/L	1.01 (0.99–1.03)	0.091
Total bilirubin, g/L	0.97 (0.96–0.98)	< 0.001
NAFLD, yes / no	2.19 (2.04–2.34)	< 0.001

Hazard ratios (HRs) for continuous measures shown for 1–SD increment.

*WC* waist circumference, *BMI* body mass index, *LDL–C* low–density lipoprotein cholesterol, *HDL–C* high–density lipoprotein cholesterol, *BUN* Blood urea nitrogen, *GFR* glomerular filtration rate, *ALT* alanine transaminase, *NAFLD* non–alcoholic fatty liver disease.

**Table 3:** Multivariate Cox analysis for transition from metabolically healthy to unhealthy phenotype over follow–up.

Variable	Metabolically healthy					
	ALL (n = 12,910)		MH–NW (n = 8478)		MH–OW/OB (n = 4432)	
	HR (95% CI)	*P* value	HR (95% CI)	*P* value	HR (95% CI)	*P* value
conversion rate, % (n)	27.0 (3486)		19.0 (1615)		42.2 (1871)	
Age, years	1.01 (1.00–1.02)	< 0.001	1.02 (1.01–1.03)	< 0.001	1.01 (1.00–1.01)	0.046
Sex, males	0.57 (0.51–0.65)	< 0.001	0.53 (0.45–0.63)	< 0.001	0.60 (0.51–0.70)	< 0.001
Education, university degree	0.78 (0.72–0.84)	< 0.001	0.81 (0.73–0.91)	< 0.001	0.75 (0.68–0.84)	< 0.001
Occupation, worker	0.87 (0.80–0.94)	< 0.001	0.87 (0.78–0.98)	0.024	0.87 (0.78–0.97)	0.013
WC, cm	1.01 (0.99–1.02)	0.234	ND		ND	
BMI, kg/m^2^	1.02 (0.99–1.04)	0.160	ND		ND	
Systolic blood pressure, mmHg	1.02 (1.01–1.03)	< 0.001	1.02 (1.01–1.03)	< 0.001	1.02 (1.01–1.03)	< 0.001
Diastolic blood pressure, mmHg	1.01 (1.00–1.02)	0.053	1.02 (1.00–1.03)	0.009	1.00 (0.99–1.01)	0.467
Current smoking	0.92 (0.85–1.01)	0.067	1.01 (0.87–1.16)	0.957	1.11 (0.99–1.23)	0.074
Regular physical activity	0.94 (0.87–1.01)	0.087	0.98 (0.88–1.10)	0.809	0.89 (0.80–0.98)	0.016
LDL cholesterol, mmol/L	1.03 (0.99–1.08)	0.132	1.09 (1.02–1.16)	0.015	0.98 (0.92–1.04)	0.473
Triglycerides, mmol/L	1.21 (1.18–1.24)	< 0.001	1.25 (1.19–1.31)	< 0.001	1.19 (1.15–1.23)	< 0.001
HDL cholesterol, mmol/L	0.33 (0.29–0.38)	< 0.001	0.28 (0.23–0.33)	< 0.001	0.44 (0.37–0.53)	< 0.001
Fasting glucose, mmol/L	1.23 (1.18–1.27)	< 0.001	1.32 (1.26–1.38)	< 0.001	1.15 (1.08–1.22)	< 0.001
Uric acid, μmol/L	1.02 (1.01–1.03)	< 0.001	1.01 (1.00–1.02)	< 0.001	1.01 (1.00–1.02)	0.030
Estimated GFR, mL/min	0.98 (0.97–9.99)	< 0.001	0.99 (0.98–1.00)	0.001	0.98 (0.97–0.99)	< 0.001
ALT, U/L	1.00 (0.99–1.01)	0.579	1.01 (1.00–1.02)	0.011	1.00 (0.99–1.01)	0.828
Total bilirubin, g/L	0.97 (0.96–0.98)	< 0.001	0.97 (0.96–0.98)	< 0.001	0.98 (0.97–0.99)	< 0.001
NAFLD, yes / no	1.12 (1.02–1.22)	0.014	1.21 (1.03–1.41)	0.017	1.14 (1.02–1.26)	0.013

Data expressed as HR (95% CI). Hazard ratios (HRs) for continuous measures shown for 1–SD increment. BMI and WC included only in analysis for all participants. The model was adjusted for baseline age and sex (male or female), education (university degree or less); occupation (worker vs. others); smoking, regular physical activity, and NAFLD (yes or not); other risk factors of continuous variables (BMI, WC, systolic blood pressure, diastolic blood pressure, LDL cholesterol, HDL cholesterol, Triglycerides, fasting glucose, uric acid, estimated GFR, ALT and total bilirubin).

*WC* waist circumference, *BMI* body mass index, *LDL–C* low–density lipoprotein cholesterol, *HDL–C* high–density lipoprotein cholesterol, *ALT* alanine transaminase, *NAFLD* non–alcoholic fatty liver disease, *ND* not determined.

### NAFLD predicts the conversion from MH to MU across different body shape phenotypes and change patterns

**Figure 2:**
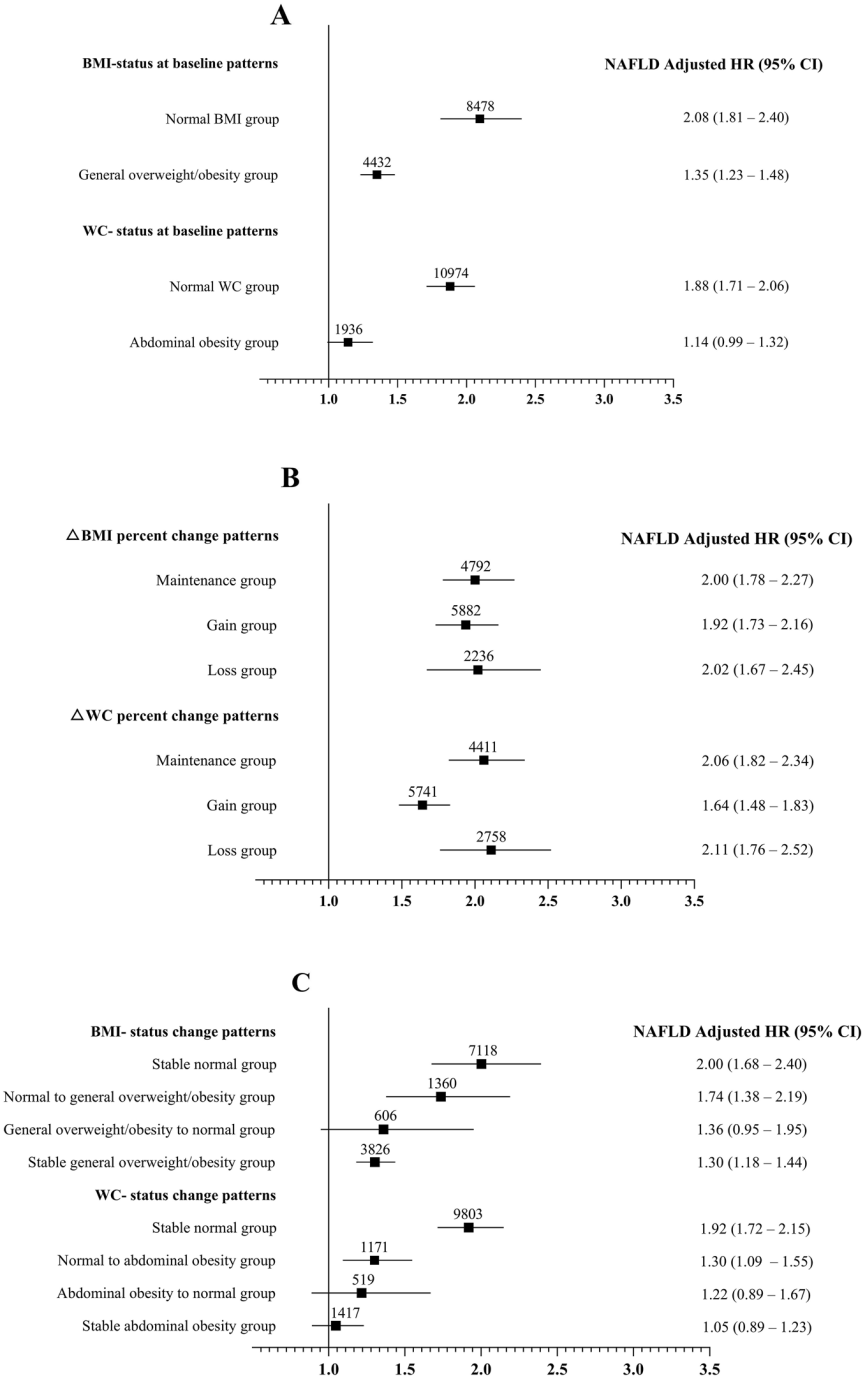
Association between the presence of NAFLD and conversion from metabolically healthy to unhealthy phenotypes according to (**A**) BMI and WC- status at baseline patterns; (**B**) ΔBMI- and ΔWC-percentage change patterns; (**C**) BMI and WC-status change patterns. Multivariate Cox analysis of the relationship between the presence of NAFLD and conversion from the metabolically healthy to unhealthy phenotype adjusted for age and sex. Values shown are the HR (95% CI) of baseline NAFLD on metabolic conversion. The values on the squares indicate the number of patients in each category. NAFLD: nonalcoholic fatty liver disease; BMI: body mass index; WC: waist circumference; HR: hazard ratio; CI: confidence interval.

Figure 2 displays the different effects of NAFLD at baseline on the conversion from the MH to the MU phenotype according to the BMI/WC categories. For BMI/WC status at baseline, the individuals in the normal BMI/WC group showed a stronger effect of NAFLD on phenotypic conversion than those in the increased BMI/WC subgroup (Fig. 2A). For △BMI/WC percent change patterns, the effects of NAFLD on phenotypic conversion were ranked in descending order: the loss, maintenance and gain subgroups (Fig. 2B). For BMI/WC status change patterns, the effects of NAFLD on phenotypic conversion were ranked in descending order: the stable normal, normal to overweight/obesity, overweight/obesity to normal, and stable overweight/obesity subgroups (Fig. 2C). The results of subgroup analysis by sex (male and female) showed subtle differences but similar trends compared with all populations (Supplemental Fig. 1). The effect of baseline NAFLD on metabolic deterioration was more pronounced among females than among males.

## Discussion

To our knowledge, the present study is one of the few studies on the risk factors for metabolic transition in the Chinese population and was the first study to examine the effect of NAFLD at baseline on metabolic transitions among different body shape change pattern subgroups. In this prospective study, we found that 27.0% of MH individuals developed metabolic abnormalities over a median follow-up of 6.9 years, and we provided clear evidence that NAFLD at baseline was an independent predictor of future conversion from the MH to the MU phenotype. Interestingly, the effect of NAFLD on metabolic deterioration differed according to body shape at baseline and body shape change patterns. Compared with non-lean NAFLD, lean NAFLD had a stronger correlation with metabolic deterioration. Notably, the effect of NAFLD on the development of metabolic abnormalities was stronger in “stable lean” individuals than in “stable non-lean” individuals and in “fluctuating non-lean” individuals than in “stable non-lean” individuals.

The transition rate from MH to MU phenotypes was 27.0% in our study, which was lower than that reported by Gao et al. in Chinese adults during 5 years of follow-up (34.90%) and close to that reported by Hwang et al. in the Korean population during 5.1 years of follow-up (23.5%)^16,26^. The transition rate from the MH to MU phenotype in different studies or different countries might be due to ethnic differences, follow-up duration and different diagnostic criteria for overweight, obesity and metabolic abnormalities. To our knowledge, few studies have investigated the metabolic transition rate of the lean population. This rate was 19.1% in our study, which provides evidence for metabolic deterioration in the lean population. Accumulating evidence has revealed that 33–52% of non-lean individuals transition from the MH to the MU phenotype over 6–20 years of follow-up; in our cohort, this rate was 42.2% ^22,27–33^. These data confirm that an MH phenotype is a transient state and indicate that assessments of metabolic status at a single time point are probably not appropriate for precise predictions of long-term disease risk regardless of non-lean or lean status, especially in the non-lean population^34,35^.

Furthermore, we found that MH-NW participants had a greater increase in BMI and WC than their MH-OW/OB counterparts, which was consistent with a previous study^26^. The reason might be the differences in adipocyte expansion capabilities between NW and OW/OB populations; NW individuals have more space for weight growth than OW/OB people, which indicates that NW individuals should also pay attention to weight control^36,37^. In contrast, higher incidences of metabolic deterioration were observed in the MH-OW/OB group than in the MH-NW group (42.2% vs. 19.1%). Similarly, Hamer et al. reported that MH-OB individuals were four times more likely to transition to the MU-OB phenotype than MH-NW individuals; Hwang et al. found that the conversion rates from the MH to the MU phenotype were 12.9%, 29.2% and 48.1% in people who were NW, overweight and obese, respectively^26,31^. Why do MH-NW individuals gain more weight than MH-OW/OB individuals, while the MH-OW/OB population is more prone to metabolic deterioration than the MH-NW population? The personal fat threshold (PFT) hypothesis proposed by Taylor et al. might serve as an explanation^36^. Each individual could have a PFT that determines their susceptibility to developing an MU phenotype in relation to their degree of β-cell function and insulin sensitivity. Gaining sufficient weight to cross their PFT would trigger the condition, whereas losing their ‘excess weight’ could return them to metabolic health. Increasing evidence has revealed that the conversion from the MH to the MU phenotype is associated with an increased risk of cardiovascular disease mortality^34,35^. Therefore, we should closely monitor the risk of cardiovascular disease in OW/OB individuals and pay more attention to weight monitoring in NW individuals.

Little is known about clinical variables that predict future metabolic conversion in the general population. Some studies have suggested that visceral abdominal fat accumulation or VAI predicts conversion in obese subjects and that NAFLD or BMI could predict conversion in NW individuals^12,16,17,26,37^. In our study, positive associations were observed between metabolic conversion and current smoking habits and individual components of MetS, and a significantly high risk of NAFLD was observed. In contrast, there were inverse associations between metabolic conversion and individuals who had a university degree, worked, engaged in regular physical activity, and had higher levels of HDL cholesterol and total bilirubin. Similarly, Hwang et al. reported that age, a current smoking habit, daily alcohol consumption, individual components of MetS (other than HDL cholesterol levels), HOMA-IR values, liver enzymes, inflammatory markers and fatty liver were associated with the conversion from the MH to the MU phenotype; on the other hand, male, regular physical activity, HDL cholesterol levels, and eGFR values were inversely associated with this conversion^26^. In general, the risk factors for and protective factors against this conversion in our study were mostly consistent with published literature^12,17,37^. We are the first to find that a university education and employment status might be inversely associated with metabolic conversion; this finding is consistent with reports by the China Obesity Survey that the burden of obesity in China is shifting to people of low economic and social status^2,38^. In addition, total bilirubin was a protective factor against metabolic deterioration in our study. Accumulating evidence indicates that bilirubin produces health benefits due to its potent antioxidant, anti-inflammatory and immunomodulatory actions^39–41^. Moderately high bilirubin levels are positively associated with a reduced risk of cardiovascular disease, diabetes, MetS and obesity^42,43^.

Notably, NAFLD was a strong predictor of metabolic conversion; this finding, along with some other studies, found that visceral abdominal fat accumulation was also related to metabolic conversion, suggesting that NAFLD, which represents the deposition of visceral fat, is more closely related to metabolic conditions than subcutaneous fat deposition^26,37^. NAFLD interacts with the regulation of multiple metabolic pathways and is bidirectionally linked with components of MetS. In 2020, a panel of international experts proposed that the new nomenclature be changed from NAFLD to metabolic dysfunction–associated fatty liver disease (MAFLD)^44^. Our findings also suggested that the term “MAFLD” provides a conceptual framework for disease diagnosis, risk stratification, and improved clinical and multidisciplinary care. However, the mechanism underlying the association between NAFLD and metabolic deterioration remains unknown. Some researchers attribute this to insulin resistance, given the association between NAFLD and insulin resistance; others support alternative conclusions^37,45–47^. Unfortunately, insulin resistance-related measures were not included in our study. We believe that systemic inflammation, oxidative stress, gut microbiota alterations and genetic risk factors may also be involved in this conversion^15^.

Furthermore, we found that the correlation between NAFLD and metabolic deterioration was stronger in the MH-NW group than in the MH-OW/OB group, which indicates that NAFLD is more sensitive to metabolic deterioration in “lean” individuals than in “non-lean” individuals. Similar results were obtained by Hwang et al. in Korea and Hashimoto et al. in Japan^26,37^. In other words, lean NAFLD was more closely related to metabolic deterioration than non-lean NAFLD. The possible explanations are as follows. Although excess adipose tissue is generally regarded as metabolically harmful, it also protects against overnutrition by serving as a buffer against metabolic risk factors. Moreover, the deficiency of adipose tissue leads to the redistribution of fat to skeletal muscle and the liver, resulting in MetS, including severe insulin resistance^48,49^. Additionally, fatty acid theory and genetic heterogeneity may also serve to explain our findings; however, these two indicators were not included in our study^47,49,50^. Notably, we explored the strength of the effect of NAFLD on metabolic conversion between different body shape change pattern subgroups. The effects of baseline NAFLD on metabolic conversion are ranked in descending order as follows: the loss, maintenance and gain subgroups. Additionally, the association between NAFLD and metabolic deterioration was stronger in the “stable lean” group than in the “stable non-lean” group, and the association was still stronger in the “fluctuating non-lean (moving from normal to overweight/obesity)” group than in the “stable non-lean” group. To our knowledge, no other study has focused on the effects of baseline NAFLD on MH conversion in different body shape change pattern subgroups. One explanation for our findings is that obese individuals often present a diverse cardiovascular profile, including high triglycerides and low HDL cholesterol levels, high blood pressure, hyperglycemia and insulin resistance. These strong associations with multiple cardiometabolic risk factors and insulin resistance might partially dilute the role of NAFLD as a predictor for metabolic conversion. On the other hand, lean NAFLD individuals presented with abnormal body fat distribution, that is, less subcutaneous fat and more visceral obesity, suggesting a higher risk of an MU phenotype. Finally, Chinese adults are reported to have a higher proportion of body fat and a greater propensity for abdominal adiposity than their Western counterparts, and visceral adiposity in the lean population might be more common in China^10,51,52^. Moreover, the effects of baseline NAFLD on metabolic conversion were stronger in females than in males. Although we were unable to clarify the underlying mechanism of these sex-based differences in this study, the difference could arise from the differential influence of sex hormones on the transition of body composition in men and women.

The prevalence of lean NAFLD has been described in different ethnic populations as follows: 10.6% in multinational cohorts, 20.0% in China, 22.1% in Japan, 33.5% in India, 12.6% in South Korea, 10.8% in the USA and 11.7% in Greece^53–59^. Lean NAFLD was not a benign condition. Younes et al. reported retrospective findings from 1,339 patients with biopsy-proven NAFLD^60^. Lean NAFLD patients may progress to metabolic comorbidities and both hepatic and extrahepatic complications, including hepatocellular carcinoma and CVD events. Hagström et al. observed 646 patients with biopsy-proven NAFLD and found that lean NAFLD patients were at higher risk for the development of future severe liver disease^61^. Another systematic review with meta-analysis showed that lean and obese individuals with NAFLD share a common altered metabolic and cardiovascular profile^62^. All these results implied that lean NAFLD subjects can develop the full spectrum of metabolic comorbidities and liver damage that occurs in non-lean NAFLD subjects. Thus, the biased BMI-driven approach for body size phenotyping in the NAFLD population may need to be reappraised. BMI does not entirely explain the association between increased body fat, compared with visceral abdominal fat, and hepatic steatosis. We recommended that WC and visceral abdominal fat analysis should be included in assessments of obesity in the Chinese population, not just weight and BMI^2^. In general, not all lean individuals are MH; thus, we should assess metabolic risk by screening for ectopic fat deposition, such as fatty liver.

Our study found that NAFLD was a strong predictor of MH deterioration in the Chinese population, especially in lean individuals. Furthermore, we explored how NAFLD predicted the risk of metabolic deterioration in different body shape change pattern subgroups and found that the association between NAFLD and metabolic deterioration was stronger in the “fluctuating non-lean” group than in the “stable non-lean” group. These findings have some highly relevant clinical implications. First, lean subjects may not all be classified as “healthy”, and they should be screened early to detect NAFLD. Second, lean NAFLD should draw considerable attention to metabolic abnormalities, and similar to non-lean individuals. Lean NAFLD patients also need to pursue proper lifestyle management, including weight control and physical activity, to prevent cardiometabolic diseases.

Our study had several limitations. First, participants’ insulin sensitivity (usually defined as a low HOMA-IR value) was not estimated in this study; such measurements can help us more precisely define metabolic health. Second, despite the large cohort size, this was not a community-based study but a health checkup-based cohort study. Our subjects were therefore not necessarily representative of the general Chinese or Asian population, so our findings should be confirmed by a multicenter cohort study. Third, although ultrasonography is widely (applied in 90.56% of all NAFLD-related studies in China) and accurately (pooled sensitivity, 84.8%; specificity, 93.6%) performed to detect fatty liver, we did not use biopsy to identify the severity of NAFLD^63^. We applied noninvasive serum biochemistry markers, such as ALT, albumin and total bilirubin levels, to preliminarily predict risk stratification. Finally, we did not evaluate hard outcomes such as mortality and major cardiovascular events associated with the conversion from the MH to the MU phenotype.

## Conclusion

In conclusion, the assessment of metabolic status at a single time point is probably not appropriate for precise predictions of long-term health risk, especially in the overweight/obese population. Lean NAFLD is not as benign as currently considered and requires more attention during screening for metabolic status. In the future, the application of precise anthropometric and metabolic phenotyping strategies could enable the proper assessment of individuals based on metabolic health profiles.

## Supplementary Information

Supplementary Information 1.

Supplementary Information 2.

## Data Availability

The datasets generated and/or analyzed during the current study are available through the journal.
