# Supplementary material for: Association between non-alcoholic fatty liver disease and metabolically healthy deterioration across different body shape phenotypes at baseline and change patterns
Source: Sci Rep. 2022 Aug 30;12:14786. doi: 10.1038/s41598-022-18988-x (PMC9427771; doi:10.1038/s41598-022-18988-x)
Supplement: Supplementary file 2 — Supplementary Information 2. [file 41598_2022_18988_MOESM2_ESM.docx]

**Supplemental Material**

**Association between non-alcoholic fatty liver disease and metabolically healthy deterioration across different body shape phenotypes at baseline and change patterns**

Liu Lei^a^, Wang Changfa^b^, Wang Jiangang^a^, Chen Zhiheng^a^, Yuan Ting^a^, Zhu Xiaoling^a^, Deng Yuling^a^, Wang Yaqin^a,*^

^a^ Health Management Center, The Third Xiangya Hospital, Central South University, No.138 Tongzipo Road, Yuelu District, Changsha, Hunan, China, 410013

^b^ General Surgery Department, The Third Xiangya Hospital, Central South University, No.138 Tongzipo Road, Yuelu District, Changsha, Hunan, China, 410013

*Corresponding Author: Wang Yaqin, MD, Health Management Center, The Third Xiangya Hospital, Central South University, No.138 Tongzipo Road, Yuelu District, Changsha, Hunan, China, 410013. Tel.: +86-0731-88618572; +86-15274810930; fax: +86-0731-88921910; E-mail address: wangy11@csu.edu.cn

**Methods**

**Definition**

The presence of type 2 diabetes and hypertension were defined according to the Chinese guidelines as previous study^[1]^. Hypertension was defined according to the following criteria: (1) systolic BP ≥140mmHg and/or diastolic BP ≥90mmHg, (2) self-reported physician-diagnosed hypertension, and/or (3) antihypertensive medication. Diabetes was defined by the presence of any of the following: (1) self-reported history of physician-diagnosed diabetes; (2) current use of insulin or oral anti-diabetic drugs, and/or (3) fasting plasma glucose ≥ 7.0 mmol/L. The diagnosis of prediabetes was based on American Dia-betes Association criteria such as FPG 5.6–6.9 mmol/l^[2]^.

**Supplemental Table 1.** The clinical characteristics of metabolic-related medical history of participants at baseline and second survey.

| **Variable** | **Metabolically healthy** | | | **p value** |
| --- | --- | --- | --- | --- |
|  | All | MH–NW | MH–OW/OB |  |
| **Number of subjects** | n = 12910 | n = 8478 | n = 4432 |  |
| **Baseline** |  |  |  |  |
| Hypertension, n (%) | 270 (2.1) | 101 (1.2) | 169 (3.8) | <0.001 |
| Diabetes, n (%) | 84 (0.7) | 45 (0.5) | 39 (0.9) | 0.020 |
| Prediabetes, n (%) | 904 (7.0) | 557 (6.6) | 347 (7.8) | 0.009 |
| Blood pressure-lowering drugs, n (%) | 177 (1.4) | 69 (0.8) | 108 (2.4) | <0.001 |
| Glucose-lowering drugs, n (%) | 32 (0.2) | 20 (0.2) | 12 (0.3) | 0.719 |
| Lipid-lowering drugs, n (%) | 29 (0.2) | 17 (0.2) | 12 (0.3) | 0.437 |
| **Resurvey** |  |  |  |  |
| Hypertension, n (%) | 504 (3.9) | 190 (2.2) | 314 (7.1) | <0.001 |
| Diabetes, n (%) | 119 (0.9) | 54 (0.6) | 65 (1.5) | <0.001 |
| Prediabetes, n (%) | 1640 (12.7) | 966 (11.4) | 674 (15.2) | <0.001 |
| Blood pressure-lowering drugs, n (%) | 412 (3.2) | 155 (1.8) | 257 (5.8) | <0.001 |
| Glucose-lowering drugs, n (%) | 81 (0.6) | 40 (0.5) | 41 (0.9) | 0.003 |
| Lipid-lowering drugs, n (%) | 100 (0.8) | 53 (0.6) | 47 (1.1) | 0.010 |

**Supplemental Figure 1.** **Association between NAFLD and conversion from metabolically healthy to unhealthy phenotype by sex categories according to (A) BMI and WC- status at baseline patterns in male; (B) △BMI- and △WC- percentage change patterns in male; (C) BMI and WC-status change patterns in male; (D) BMI and WC- status at baseline patterns in female; (E) △BMI- and △WC- percentage change patterns in female; (F) BMI and WC-status change patterns in female.**

Multivariate Cox analysis for relationship between NAFLD and conversion from metabolically healthy to unhealthy phenotype adjusted for age. Values shown are the HR (95% CI) for the effect of baseline NAFLD on metabolic conversion. The values on the squares indicate number of cases in each category. NAFLD, nonalcoholic fatty liver disease; BMI, body mass index; WC, waist circumference; HR, hazard ratio; CI, confidence interval.

**References**

[1] Y. Lu, R. Pechlaner, J. Cai, et al. Trajectories of Age-Related Arterial Stiffness in Chinese Men and Women[J]. J. Am. Coll. Cardiol. 2020, 75(8), 870-880. DOI: 10.1016/j.jacc.2019.12.039.

[2] Summary of Revisions: Standards of Medical Care in Diabetes-2019[J]. Diabetes Care. 2019, 42(Suppl 1), S4-S6. DOI: 10.2337/dc19-Srev01.
